# Mapping Functions in Health-Related Quality of Life: Mapping from Two Cancer-Specific Health-Related Quality-of-Life Instruments to EQ-5D-3L

**DOI:** 10.1177/0272989X15587497

**Published:** 2015-10

**Authors:** Tracey A. Young, Clara Mukuria, Donna Rowen, John E. Brazier, Louise Longworth

**Affiliations:** School of Health and Related Research (ScHARR), University of Sheffield, Sheffield, UK (TAY, CM, DR, JEB); HERG, Brunel University, Uxbridge, Middlesex, UK (LL)

**Keywords:** health-related quality of life, mapping functions, cancer, EQ-5D-3L, FACT-G, EORTC-QLQ-C30

## Abstract

**Background.** Clinical trials in cancer frequently include cancer-specific measures of health but not preference-based measures such as the EQ-5D that are suitable for economic evaluation. Mapping functions have been developed to predict EQ-5D values from these measures, but there is considerable uncertainty about the most appropriate model to use, and many existing models are poor at predicting EQ-5D values. This study aims to investigate a range of potential models to develop mapping functions from 2 widely used cancer-specific measures (FACT-G and EORTC-QLQ-C30) and to identify the best model. **Methods.** Mapping models are fitted to predict EQ-5D-3L values using ordinary least squares (OLS), tobit, 2-part models, splining, and to EQ-5D item-level responses using response mapping from the FACT-G and QLQ-C30. A variety of model specifications are estimated. Model performance and predictive ability are compared. Analysis is based on 530 patients with various cancers for the FACT-G and 771 patients with multiple myeloma, breast cancer, and lung cancer for the QLQ-C30. **Results.** For FACT-G, OLS models most accurately predict mean EQ-5D values with the best predicting model using FACT-G items with similar results using tobit. Response mapping has low predictive ability. In contrast, for the QLQ-C30, response mapping has the most accurate predictions using QLQ-C30 dimensions. The QLQ-C30 has better predicted EQ-5D values across the range of possible values; however, few respondents in the FACT-G data set have low EQ-5D values, which reduces the accuracy at the severe end. **Conclusions.** OLS and tobit mapping functions perform well for both instruments. Response mapping gives the best model predictions for QLQ-C30. The generalizability of the FACT-G mapping function is limited to populations in moderate to good health.

## Introduction

In the United Kingdom, the National Institute for Health and Care Excellence (NICE) recommend the EQ-5D to measure preference-based health-related quality of life (HRQL) to estimate quality-adjusted life years (QALYs) in economic evaluations.^1^ It has been demonstrated that the EQ-5D-3L is sensitive to changes in HRQL in patients with cancer.^2^ However, many cancer studies do not include the EQ-5D and are more likely to include 1 of 2 cancer-specific HRQL questionnaires: the European Organization for Research and Treatment Quality of Life Questionnaire Core 30 (EORTC QLQ-C30) or the Functional Assessment of Cancer Therapy—General Scale (FACT-G), both of which are non-preference-based measures. NICE guidelines acknowledge that the EQ-5D is not always available and in these situations recommend using mapping to estimate EQ-5D. Mapping allows health state utility values to be predicted when no preference-based measure is included in the study. This approach involves estimating the relationship between a non-preference-based measure and a generic preference-based measure using statistical association, and it requires a degree of overlap between the descriptive systems of the 2 measures and that the 2 measures are administered on the same population. A review of mapping functions by Brazier et al.^3^ demonstrates that researchers use a number of models, including ordinary least squares regression (OLS), generalized linear models, tobit, censored absolute deviance (CLAD), 2-part models, and response mapping, to predict health state preference values. Studies also report a variety of methods to assess model and predictive performance, including predicted mean and standard deviation, median, range of predictions, Akaike information criteria (AIC), Bayes information criteria (BIC), *R*^2^, pseudo-*R*^2^, mean estimates across severity groups, root mean square error (RMSE), mean square error (MSE), and mean absolute error (MAE).

Only 1 mapping function has been published that maps from FACT-G to EQ-5D-3L: it fits separate OLS and CLAD models at the FACT-G dimension level, and shows that values are poorly predicted for high and low EQ-5D values.^4^ There are several published mapping functions for the EORTC QLQ-C30. Potentially, the most useful function is that of McKenzie and van der Pol,^5^ who used OLS to predict EQ-5D-3L values and ordered probit to predict EQ-5D-3L dimension levels. The ordered probit model did not produce reliable predictions, but the OLS gave reasonable EQ-5D-3L estimates. It is possible that other mapping functions such as the tobit model would produce more accurate estimates, although these are not explored in this article. Recently, Khan and Morris^6^ explored a number of alternative models for predicting EQ-5D in patients with lung cancer; their models assume EQ-5D scores lie between 0 and 1, and they show that the nonlinear beta-binomial model gave the best predictions of EQ-5D. The beta-binomial model is not going to be applicable to all populations because some respondents will have negative EQ-5D scores. Proskorovsky et al.^7^ used linear regression to predict EQ-5D-5L using a sample of 154 patients with multiple myeloma and to estimate models with and without the EORTC QLQ-MY20; this is used in conjunction with the EORTC QLQ-C30 to assess quality of life in patients with multiple myeloma, but the results are not validated using an external sample. The remaining published mapping functions are not as useful. Kontodimopoulous et al.^8^ used OLS to predict EQ-5D-3L; the model is based on a small sample of patients, and they state that their function is unreliable. Wu et al.^9^ required data on FACT-G as well as EORTC QLQ-C30 to produce mapping estimates, which studies may not routinely collect together. Pickard et al.^10^ mapped to patient time trade-off (TTO) values rather than the EQ-5D-3L index. Crott and Briggs^11^ developed their function from a female-only sample, and therefore the results are not generalizable, as evident by results from assessment of this mapping function^12^ that demonstrate that EQ-5D-3L estimates are not stable among different data sets. Versteegh et al.^13^ only used OLS in their analysis and only provided Dutch values, not UK ones; whereas Versteegh et al.^14^ developed separate mapping functions for those in poor health versus those in better health, and these have limited reliability due to small sample sizes at the *poor health* end. Some of these mapping functions have worse predictive performance than the McKenzie and van der Pol^5^ model in a multiple myeloma patient data set.^15^ There are other papers that map to non-UK values of the EQ-5D-3L.^16–18^

Given the lack of robust mapping studies in this area, the aim of this article is to estimate mapping functions from 2 cancer-specific HRQL measures, FACT G and EORTC QLQ-C30, to the EQ-5D-3L to test the applicability of different mapping approaches that have been used in the literature and provide recommendations for future mapping studies. This article presents the results from testing alternative common modeling techniques that are recommended in the literature and uses recommended criteria to identify the most appropriate mapping functions.

EQ-5D-3L is referred to as EQ-5D and EORTC QLQ-C30 as QLQ-C30 in the rest of the article.

## Methods

### Measures

#### EQ-5D

The EQ-5D is the most widely used generic preference-based measure of health-related quality of life. The EQ-5D has 5 dimensions: mobility, self-care, usual activities, pain/discomfort, and anxiety/depression.^19^ Each dimension has 3 levels of severity. Each health state described by the EQ-5D has a utility value anchored on a 0 to 1 scale, where 0 represents death and 1 represents full health. The values used here are produced using the UK value set.^20^

#### FACT-G

The FACT-G is a 27-item cancer-specific HRQL measure that has been widely validated.^21^ Each item has 5 options ranging from *not at all* (a score of 0) to *very much* (a score of 4), and these are summed to obtain a global score as well as 4 subscale scores: physical well-being, social/family well-being, emotional well-being, and functional well-being.

#### EORTC QLQ-C30

The QLQ-C30 is a 30-item cancer-specific HRQL measure that has also been widely validated.^22^ Two items ask about overall quality of life and overall health, and the remainder cover 5 functioning scales (physical, role, social, emotional, and cognitive functioning) and 9 symptoms scales (fatigue, nausea and vomiting, pain, dyspnea, sleep disturbance, appetite loss, constipation, diarrhea, and financial impact).

### Data

Four data sets are used in this analysis: One contains the FACT-G and EQ-5D, and the remaining three contain the QLQ-C30 and EQ-5D and are combined to produce a reliable mapping function. The FACT-G data set consists of 530 US respondents with 13 different types of stage III and IV cancers who completed the EQ-5D and FACT-G.^23^ Fifty-two percent of respondents are male, and the average age of the sample is 59 years. The 3 data sets combined for the QLQ-C30 mapping analysis are a randomized controlled trial of 572 patients with multiple myeloma (VISTA study; ClinicalTrials.gov number NCT00111319),^24^ and 100 patients with breast cancer and 99 patients with lung cancer having consultations at a Canadian cancer clinic (Vancouver Cancer Clinic data). This gives a total of 771 cases for the mapping study; 44% of responders are male, and the mean age of patients is 68 years.

### Analysis

#### Models

Five commonly applied alternative types of model are fitted to the data: OLS, tobit, 2-part models, and splining to map to EQ-5D values, and response mapping to map to individual EQ-5D dimension scores. The most commonly used mapping model reported in the literature is OLS.^25,26^ These models are typically able to predict the mean values but are poor at predicting those in poor health and full health, and they do not allow for the fact that the EQ-5D is bounded at 1 for full health and −0.594 for the worst possible state described by EQ-5D. Tobit models are therefore fitted to allow for the bounded nature of EQ-5D, thus limiting predictions to within a credible range. An alternative model that can be fitted in an attempt to predict responders in perfect health is the 2-part model, which uses a combination of 2 different model types to predict different parts of the distribution of the data. Logistic regression is fitted to predict the probabilty of whether responders are in full health (FH), and a truncated OLS is applied to predict EQ-5D values for those not in full health. The results from the 2 parts of the model are combined to obtain an overall value using an expected value approach,^27^ that is,

$$\begin{array}{l} \mathrm{Expected}\left(\mathrm{EQ}-5D\right)=\left(\mathrm{Probability}\left(\mathrm{Full health}\right)*\left(\mathrm{EQ}-5\mathrm{D in FH}\right)\right)\\ +\left(\mathrm{EQ}-5\mathrm{D if not FH}*\left(1-\mathrm{Probility}\left(\mathrm{Full health}\right)\right)\right) \end{array}$$

Given that the EQ-5D fails to approximate to the normal distribution, the final model that is fitted to the EQ-5D index uses splining, also known as *fractional polynomials*. Splining can be used to identify changes (cut points) in the distribution of the continuous explanatory variables (QLQ-C30 or FACT-G total or domain scores) and models these changes using different mathematical functions. The cut points were identified using the multivariable fractional polynomial function in Stata.^28^ This function identifies cut points and fits all possible polynomial functions to the data using power functions ranging from −2 to 3, and identifies the best-fitting model for predicting the outcome variable (EQ-5D score). Splining functions are applied to the best-fitting OLS/tobit dimension based models to test whether splines offered an improvement over using squared terms.

In the mapping literature, OLS, tobit, 2-part, and splining models are usually reliable at predicting the group EQ-5D mean and median values, and they are able to distinguish between severity levels but are poor at predicting the overall range of EQ-5D values. An alternative to modeling the EQ-5D value is to use response mapping, which can predict the 5 EQ-5D dimension levels.^29,30^ Multinomial logistic regression models are estimated for each dimension, and the estimates from these regressions are used to categorize respondents into levels 1, 2, or 3 of each of the EQ-5D dimensions and thus predict the EQ-5D health state for each respondent. A total of 2000 Monte Carlo simulations are run to estimate EQ-5D health states. The standard set of UK general population values is then applied to each predicted health state to obtain EQ-5D values.^20^

Eight model specifications (models 1 to 8) are fitted for OLS, tobit, and 2-part models; these specifications can be seen in Table 2, illustrated using the FACT-G data set. Model 1 uses the FACT overall score. Models 2 to 5 are based on FACT-G domain scores; model 2 includes all domains regardless of statistical significance, model 3 includes only statistically significant domains, model 4 includes squared and square root terms, and model 5 includes interaction terms. Models 6 and 7 are item-level models that include only significant items; model 7 merges item levels for levels that are shown to be disordered in model 6 (item coefficent size does not increase or decrease by item level). Patient and disease characteristics were explored in model 8. Splining models were fitted to total score and domain level (models 1 to 5).

To avoid overfitting models, the rule of 10 participants per variable for continuous models and 10 events for the smallest category for response-mapping models is used. The FACT-G data set does not include responders with very poor health, with only 0.9% of responders having negative EQ-5D values in contrast to 18% of responders reporting full health on the EQ-5D. No responder had extreme problems for mobility, and few responders indicated extreme problems for self-care (0.4%), usual activities (6%), pain/discomfort (3%), or anxiety/depression (2%). Applying the overfitting rule to the response-mapping EQ-5D level 3 predictions for models that would include FACT-G item levels (models 6 and 7) restricted the number of items that should be included to 1 item. A model that could include only 1 FACT-G item is not going to be useful at predicting EQ-5D dimension responses, so model 6 and 7 are not fitted. There were a slightly higher number of EQ-5D level 3 responders for the QLQ-C30 data set, but we were again restricted to including 1 QLQ-C30 item; again, we chose not to include model 6. However, because there are more EQ-5D level 3 responses, it was possible to collapse QLQ-C30 items into a smaller number of levels. Therefore, we were able to fit model 7 to the QLQ-C30 data set.

Models are fitted using backward regression, and variables are removed from the model if nonsignificant at *p* < 0.1. When variables are highly correlated (correlation > 0.7), the variable that is most significant and judged most likely to map to the EQ-5D based on prior expectations is selected. Standard errors of regression coefficents are calculated from bootstrap estimates with 5000 bootstrap samples for each model.

Model goodness of fit is measured using AIC, BIC, and MAE, in which smaller values indicate better model fit. Model performance is also assessed visually by plotting observed and predicted EQ-5D values. Standard model tests are also examined, including *R*^2^ and adjusted *R*^2^ for OLS and pseudo *R*^2^ for the other models; the Ramsey Regression Equation Specification Error Test (RESET) is used in OLS to test whether nonlinear conbinations of variables in the model help explain the variability, where a significant result indicates that a nonlinear model is more appropriate. Sigma is reported for tobit and truncated regression models, and is the equivalent to RMSE in linear regression models. The link test is used to check model specification. The Hosmer–Lemeshow test is used to assess goodness of fit for logistic regression models (first part of 2-part models), which assesses whether predicted probabilities agree with observed probabilities and should be nonsignificant for a model that accurately predicts observed values.

#### Model performance and discrimination

Summary statistics, including mean and range, are examined to assess overall model predictions. A severity measure is used to assess the discriminative performance of the predicted EQ-5D value among different severity groups. For FACT-G, the Eastern Cooperative Oncology Group (ECOG) performance status^31^ is used to categorize respondents according to severity. The ECOG has 5 response categories: normal activity without symptoms, some symptoms but do not require bed rest during the waking day, require bed rest for less than 50% of the waking day, require bed rest for more than 50% of the waking day, and unable to get out of bed. There are no patients in the most severe level, and few patients (*n* = 21 [4%]) required bed rest for more than 50% of the waking day; therefore, this category is merged with *do not require bed rest less than 50% of the waking day*. The general health status item of the QLQ-C30 is used to categorize respondents according to severity in the QLQ-C30 data set. Response options ranged from poor to excellent (i.e., from 1 to 7). Discriminative ability among severity groups using these measures is tested using ANOVA. MAEs are reported for each subgroup.

#### Model validation

Models are validated internally using bootstrapping techniques to estimate a shrinkage factor that allows for overoptimism of the predictive ability of the fitted model (a model is better at predicting estimates on the same data from which the model is derived, compared to an external data set). Methods reported by Steyerberg et al.^32^ are used to assess all models, and shrinkage coefficients are reported to counter overoptimism of estimates. To estimate the shrinkage factors, 5000 bootstrap estimates are run, and for each bootstrap sample the EQ-5D predicted score (linear prediction) is calculated. The slope of the EQ-5D predicted score in relation to the observed score is then calculated for each sample, and the mean slope across the 5000 samples denotes the shrinkage coefficient. A shrinkage coefficient of less than 1 (the typical value expected for a shrinkage coefficient) reflects an “overfitting” of the data.

#### Model selection

When producing a mapping model, the factors that are important in selecting a model are accuracy of the predicted mean and standard error, the range of predictions, MAE, shrinkage, and the reproducibility of the model among different severity states. Mapping and model-fitting literature do not suggest a single criterion for use in selecting the best-fitting model, and the most appropriate measure may depend on the purpose of the mapping function; for example, populating a model may require accurate predictions of mean preference-based values for different severity groups, whereas accurate overall means at different time points may be sufficient when subgroup analysis is not undertaken. Therefore, when selecting models, all criteria are given equal weighting, models are ranked based on these factors, and the mean rank per model is estimated. The model with the best ranking is then selected, and these are then compared among the different estimation methods (OLS, tobit, 2-part, splining, and response mapping). All mapping functions are fitted in STATA version 12.^33^

Financial support for this study was provided entirely by a grant from the MRC-NIHR (UK Medical Research Council and National Institute for Health Research) Methodology Research Programme. The study was part of the NICEQoL project looking at the use of generic and condition-specific measures of HRQL for NICE decision making.^2^ The funding agreement ensured the authors’ independence in designing the study, interpreting the data, writing, and publishing the report.

## Results

### Descriptive Statistics

#### FACT-G

**Figure 1:**
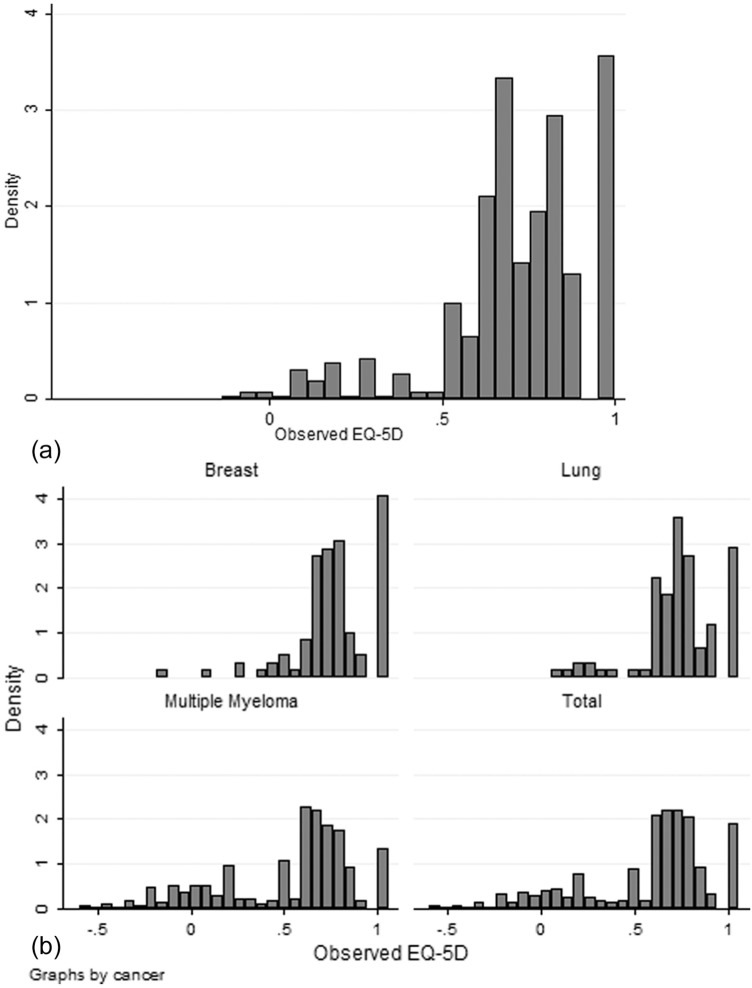
EQ-5D distribution for the (a) Functional Assessment of Cancer Therapy—General Scale (FACT-G) data set; and (b) European Organization for Research and Treatment Quality of Life Questionnaire Core 30 (QLQ-C30) data sets.

The characteristics and a summary of EQ-5D values and FACT-G responses are presented in Table 1. EQ-5D values did not cover the full possible range and went from −0.135 to 1. The distribution of the EQ-5D index for the FACT-G data set is shown in Figure 1a, which reflects the distribution of possible EQ-5D values. The average global FACT-G score ranges from 33 to 108; thus, like the EQ-5D, it did not cover the worse end of the FACT-G scale. The relationship between the global FACT-G score and EQ-5D is moderate (Spearman’s correlation ρ = 0.575). The EQ-5D correlates moderately with the physical and functional domains of the FACT-G (ρ = 0.566, ρ = 0.501, respectively), although the correlations are weak for the social and emotional domains (ρ = 0.178, ρ = 0.382, respectively).

**Table 1:** Characteristics of the FACT-G and QLQ-C30 Data Sets

	FACT-G Data Set		QLQ-C30 Data Sets							
	(*n* = 530)		All (*n* = 771)		Breast (*n* = 100)		Lung (*n* = 99)		Multiple Myeloma (*n* = 572)	
	Mean	SD	Mean	SD	Mean	SD	Mean	SD	Mean	SD
Age, y	59.01	11.92	68.31	9.56	53.9	10.94	62.73	10.5	71.79	5.45
Male, %	51.7		44.1		0.0		48.0		50.0	
**EQ-5D-3L**										
EQ-5D utility value	0.721	0.22	0.58	0.342	0.765	0.20	0.742	0.20	0.519	0.36
EQ-5D = 1, %	17.5		10.7		24.0		17.1		7.9	
Range of EQ-5D-3L	−0.135 to 1		−0.594 to 1		−0.144 to 1		0.088 to 1		−0.594 to 1	
**FACT-G**										
Physical	20.16	5.70	—	—	—	—	—	—	—	—
Social	22.68	4.77	—	—	—	—	—	—	—	—
Emotional	17.50	4.46	—	—	—	—	—	—	—	—
Functional	17.58	5.86	—	—	—	—	—	—	—	—
Total score	77.92	15.16	—	—	—	—	—	—	—	—
**EORTC QLQ-C30**										
Physical functioning	—	—	64.81	25.59	78.27	19.87	69.76	19.62	61.6	26.5
Role functioning	—	—	59.14	33.18	72.67	27.68	67.51	26.98	55.33	34.17
Emotional functioning	—	—	69.71	24.91	73	22.69	76.26	21.47	68.01	25.61
Cognitive functioning	—	—	76.05	22.74	76.83	22.83	77.27	20.54	75.7	23.11
Social functioning	—	—	69.13	29.82	72	26.15	73.74	23.82	67.83	31.25
Fatigue‡	—	—	45.42	26.16	39.11	20.86	42.87	23.11	46.97	27.3
Nausea‡	—	—	9.014	17.87	11	19.85	10.27	16.79	8.45	17.69
Pain‡	—	—	40.4	32.99	23	24.25	22.56	23.49	46.53	33.54
Dyspnea‡	—	—	24.73	28.97	16.67	22.47	36.7	30.67	24.07	29.09
Sleep disturbance‡	—	—	32.68	32.6	34	31.06	30.98	28.27	32.75	33.59
Appetite loss‡	—	—	27.37	32.53	19.67	28.46	28.62	32.3	28.5	33.1
Constipation‡	—	—	23.13	30.71	11.67	23.39	22.9	29.98	25.17	31.56
Diarrhea‡	—	—	9.511	19.86	15.67	26.99	11.45	20.29	8.1	18.04
Financial impact‡	—	—	19.76	28.78	23.67	30.45	22.9	28.83	18.53	28.42
Global quality of life	—	—	52.76	23.18	67.92	18.17	62.12	21.04	48.48	22.75

For FACT-G, higher scores indicate better well-being and quality of life. The EORTC QLQ-C30 dimension score ranges from 0 to 100; higher scores indicate better functioning and quality of life.

FACT-G, Functional Assessment of Cancer Therapy—General Scale; QLQ-C30, European Organization for Research and Treatment Quality of Life Questionnaire Core 30.

‡ Higher scores for symptom scales indicate worse symptoms.

#### QLQ-C30

The characteristics and a summary of EQ-5D values and QLQ-C30 responses are presented in Table 1 for the combined sample and for each data set. Mean age and gender distribution varied by data set, as did mean EQ-5D values, which are lowest for the multiple myeloma data set. Only the multiple myeloma data set covered the entire range of the EQ-5D, and it has lower ceiling effects than the other data sets, with 8% of responses at full health on EQ-5D in comparison to 24% and 17% for the breast and lung cancer data sets, respectively. Figure 1b presents the histograms for each data set and the combined data set, showing that the distributions differed by data set, but without further information it was not possible to conclude whether this is due to differences in the severity of the patients in each data set or differences in the pattern of EQ-5D by condition. The scores for the QLQ-C30 scales most noticeably varied among the 3 data sets for physical functioning, role functioning, pain, dyspnea, constipation, and global quality of life. Assessment of the correlations between the EQ-5D and the QLQ-C30 scale scores indicated that the highest correlations are between physical functioning, role functioning, fatigue, and pain (ρ = 0.701, ρ = 0.688, ρ = −0.625, and ρ = −0.735, respectively).

### Mapping Results

#### Selecting models

Table 2 illustrates the model selection process for OLS. Model 8 included patient and disease characteristics, but these are not statistically significant predictors of EQ-5D score; therefore, the results for this model are not shown in Table 2. By definition, all models predict the overall mean EQ-5D value for the data set, but underestimate those in near-full or full health and overestimate those in poorer health states. No model predicts a value lower than 0.155 (observed values range from −0.135 to 1). All models are able to discriminate between different levels of health, as measured by categorized EQ-5D value and ECOG. MAE is large for those in poor health, which is expected given the range of model predictions. Item-level models consistantly performed better than the domain and total score models, although they are most likely to overfit and are poor at predicting values away from the overall mean for the data set that is similar to that of other mapping studies in the literature.^14,34^ All of the summary statistics and model performance tests are ranked. Giving all performance statistics equal weighting indicates that model 6 (significant FACT-G items) is the best-performing OLS model for estimating EQ-5D values from the FACT-G. This process is then repeated for tobit, 2-part, splining, and response mapping (see Longworth et al.^2^ for these results) for the FACT-G and the QLQ-C30. The best-ranked functions for each model (OLS, tobit, etc.) are then compared using the same approach.

**Table 2:** Summary of Observed and Predicted Values and Model Performance Statistics per FACT-G OLS Models

			OLS Model 1		OLS Model 2		OLS Model 3		OLS Model 4		OLS Model 5		OLS Model 6		OLS Model 7	
		Observed Values	Total Score		Domain Scores		Significant Domains		Significant Domains and Squared Terms		Significant Domains, and Squared and Interaction Terms		Item Levels—Significant Levels Only		Item Levels—Significant Levels Only, Collapse Unordered Items	
Mean (SD)		0.721 (0.223)	0.721 (0.128)		0.721 (0.138)		0.721 (0.138)		0.721 (0.144)		0.721 (0.146)		0.721 (0.163)		0.721 (0.161)	
Median		0.735	0.730		0.735		0.735		0.738		0.744		0.755		0.750	
Range		−0.135 to 1	0.319 to 0.975		0.357 to 0.971		0.357 to 0.972		0.198 to 0.981		0.161 to 0.946		0.115 to 0.962		0.169 to 0.961	
*R*^2^			0.331		0.383		0.383		0.417		0.432		0.535		0.524	
Adjusted *R*^2^			0.330		0.378		0.379		0.413		0.425		0.513		0.507	
AIC			−298.40		−335.20		−337.12		−365.34		−374.98		−445.43		−443.38	
BIC			−289.86		−313.84		−320.11		−343.97		−345.07		−338.60		−357.92	
Ramsey RESET			F_3, 525_ = 3.19 (*p* = 0.024)		F_3,522_ = 0.83 (*p* = 0.477)		F_3,525_ = 0.84 (*p* = 0.471)		F_3,524_ = 2.96 (*p* = 0.032)		F_3,521_ = 2.06 (*p* = 0.104)		F_3,502_ = 0.72 (*p* = 0.539)		F_3,507_ = 1.17 (*p* = 0.320)	
MAE			0.129		0.126		0.126		0.124		0.122		0.111		0.112	
Shrinkage			1.005		0.992		0.996		0.995		0.991		0.850		0.909	
		Observed Values	OLS 1		OLS 2		OLS 3		OLS 4		OLS 5		OLS 6		OLS 7	
Model Validation	n	Mean	Mean	MAE	Mean	MAE	Mean	MAE	Mean	MAE	Mean	MAE	Mean	MAE	Mean	MAE
ECOG																
Normal; no symptoms	122	0.865	0.816	0.111	0.834	0.096	0.834	0.096	0.843	0.097	0.840	0.097	0.846	0.087	0.8464	0.0870
Some symptoms	256	0.722	0.728	0.122	0.733	0.124	0.733	0.124	0.726	0.123	0.728	0.121	0.732	0.108	0.7319	0.1087
Require some bed rest	152	0.606	0.634	0.157	0.612	0.154	0.612	0.154	0.615	0.147	0.614	0.145	0.603	0.135	0.6032	0.1343
ANOVA		F_2,527_ = 55, *p* < 0.001	F_2,527_ = 92, *p* < 0.001		F_2,527_ = 134, *p* < 0.001		F_2,527_ = 135, *p* < 0.001		F_2,527_ = 125, *p* < 0.001		F_2,527_ = 117, *p* < 0.001		F_2,527_ = 107, *p* < 0.001		F_2,527_ = 108, *p* < 0.001	
Mean ranking			5.53		4.53		4.12		3.76		3.53		2.12		2.53	

There are no significant patient characteristcs for PLS, so model 8 is not presented here.

AIC, Akaike information criteria ; BIC, Bayes information criteria; ECOG, Eastern Cooperative Oncology Group; FACT-G, Functional Assessment of Cancer Therapy—General Scale; MAE, mean absolute error; OLS, ordinary least squares; PLS, partial least squares; RESET, Ramsey Regression Equation Specification Error Test.

#### Best-fitting models: FACT-G

**Figure 2:**
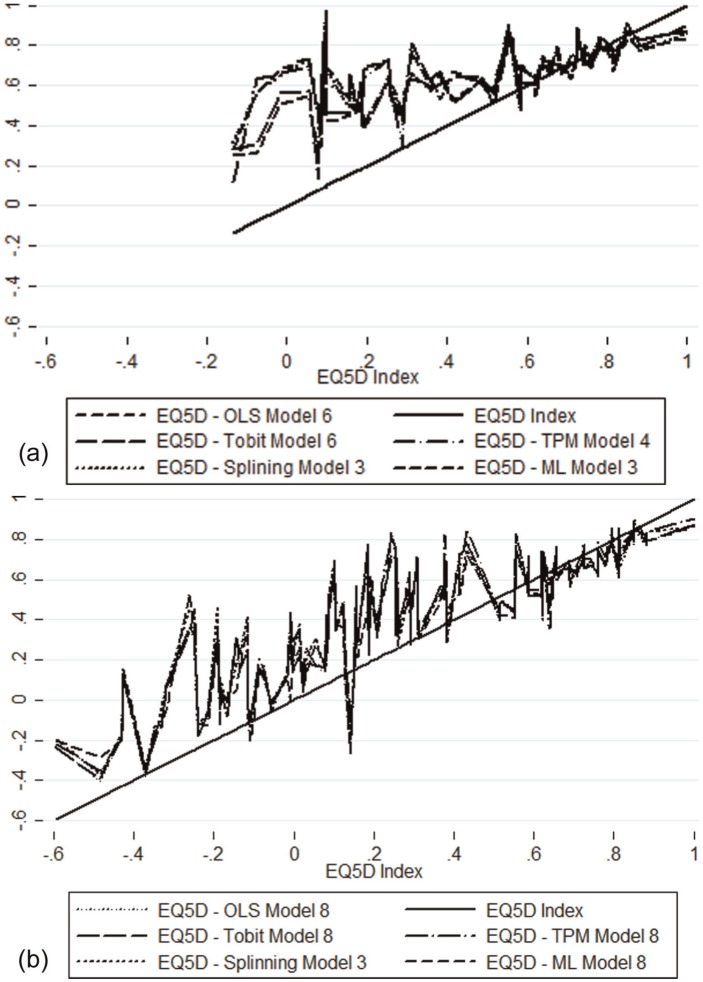
Observed and predicted EQ-5D values for the best-performing models for the (a) Functional Assessment of Cancer Therapy—General Scale (FACT-G) data set; and (b) European Organization for Research and Treatment Quality of Life Questionnaire Core 30 (QLQ-C30) data sets.

Table 3 and Figure 2 summarize the best-fitting OLS, tobit, 2-part modeling, splining, and response-mapping models for the FACT-G. FACT-G item-level models give the best model predictions for OLS and tobit, whereas a significant domain-level model with square terms is the best model for the 2-part models (model 4). Only domain levels are fitted for splining and response mapping, and model 3, which is the one with significant domains, is the best for both of these models. OLS gives the best estimates of the overall mean and the mean by severity group, and has 1 of the 2 best ranges of predicted values (the 2-part model covers the widest range). OLS was the poorest at predicting the median and had the lowest shrinkage factor, suggesting it would be the most likely to overpredict results in other studies applying the mapping algorithm. The response-mapping model gave reasonable estimates of the mean and median, but the poorest MAE among severity groups. All models failed to predict anyone in perfect health, underpredicted the top of the EQ-5D scale, and overpredicted the bottom end of the scale. However, the overprediction at the lower end of the scale is perhaps unsurprising given that few responders in the FACT-G data set reported severe problems. A mean ranking of models among the different model performance statistics shows OLS to give the best predictions (mean ranking = 2.1), followed by tobit (mean = 2.4), with 2-part models and response mapping giving the poorest predictions (mean = 3.6, mean = 3.5, respectively). Table 4 presents the regression coefficients for the best-fitting model (OLS FACT-G significant items).

**Table 3:** Summary of Observed EQ-5D Values and Ranking of Model Performance Statistics for the Best-Performing FACT-G Models

			OLS Model 6		Tobit Model 6		2-Part Model 4		Splining Model 3		ML Model 3	
		Observed Values	Significant Item Levels		Significant Item Levels		Significant Domain Scores, and Squared and Square Root Terms		Significant Domain Scores		Significant Domain Scores	
Mean (SD)		0.721 (0.223)	0.721 (0.163)		0.723 (0.161)		0.739 (0.154)		0.723 (0.144)		0.720 (0.133)	
Median		0.735	0.755		0.738		0.753		0.736		0.737	
Range		−0.135 to 1	0.115 to 0.962		0.132 to 0.957		0.119 to 0.993		0.312 to 0.974		0.268 to 0.934	
MAE			0.111		0.181		0.120		0.198		0.125	
Shrinkage			0.850		0.962		0.944		0.982		1.019	
Model Validation	N	Mean	Mean	MAE	Mean	MAE	Mean	MAE	Mean	MAE	Mean	MAE
ECOG												
Normal; no symptoms	122	0.8645	0.8464	0.0878	0.8498	0.0878	0.8302	0.0896	0.8460	0.097	0.7933	0.1009
Some symptoms	256	0.7219	0.7318	0.1080	0.7320	0.1108	0.7359	0.1211	0.7277	0.121	0.7201	0.1219
Require some bed rest	152	0.6055	0.6033	0.1353	0.6074	0.1365	0.6713	0.1410	0.6152	0.148	0.6601	0.1485
ANOVA		F_2,527_ = 55, p < 0.001	F_2,527_ = 107, p < 0.001		F_2,527_ = 109, p < 0.001		F_2,527_ = 122, p < 0.001		F_2,527_ = 130, p < 0.001		F_2,527_ = 120, p < 0.001	
Mean rank			2.08		2.42		3.58		3.25		3.5	

ECOG, Eastern Cooperative Oncology Group; FACT-G, Functional Assessment of Cancer Therapy — General Scale; MAE, mean absolute error; ML, multinomial logistic regression; OLS, ordinary least squares.

**Table 4:** Coefficients for the Best-Fitting Mapping Model From FACT-G: OLS Model 6

Domain	Item	Item Level	OLS Model 6
Physical	Lack of energy	Very much (baseline level)	
		Quite a bit	0.045
		Somewhat	0.036
		A little bit	0.071
		Not at all	0.118
	Trouble meeting need of family	Very much (baseline level)	
		Quite a bit	−0.028
		Somewhat	0.049
		A little bit	0.088
		Not at all	0.098
	Pain	Very much (baseline level)	
		Quite a bit	0.125
		Somewhat	0.219
		A little bit	0.240
		Not at all	0.342
Emotional	I feel sad	Very much (baseline level)	
		Quite a bit	−0.085
		Somewhat	−0.019
		A little bit	−0.006
		Not at all	−0.004
	Losing hope	Very much (baseline level)	
		Quite a bit	−0.081
		Somewhat	−0.007
		A little bit	0.013
		Not at all	0.060
Functional	Able to work	Not at all (baseline level)	
		A little bit	0.113
		Somewhat	0.130
		Quite a bit	0.150
		Very much	0.152
Constant			−0.597

FACT-G, Functional Assessment of Cancer Therapy—General Scale; OLS, ordinary least squares.

#### Best-fitting models: QLQ-C30

Seven models’ specifications (models 2 to 8; model 1 was excluded because the QLQ-C30 does not have an overall total score) are fitted for the QLQ-C30. Table 5 and Figure 2 present the predicted EQ-5D values for the best-fitting models for each estimation technique alongside model performance statistics for the QLQ-C30. As with FACT-G, the item-level models give the best model predictions for OLS and tobit models (model 8, including items and sociodemographic characteristics). These models are best at predicting the overall mean EQ-5D value. Item-level models with sociodemographic characteristics give the best model performance for 2-part models. The 2-part model resulted in a more accurate prediction of the median than predictions for OLS, tobit, splining, and response mapping. The splining model has the least deviation from the shrinkage coefficient of 1 (model 3). The best-performing response-mapping model includes all domains with age and gender for some of the dimensions, and this model has the lowest MAEs on average. None of the models predicts the full range of observed EQ-5D values, with no predictions at the best or worst EQ-5D values. The mean ranking indicates that the response mapping was the best-performing model (mean rank = 2.4), with OLS and tobit also performing well (mean = 2.6, mean = 2.8, respectively) and splining giving the poorest overall performance (mean = 3.7). Table 6 presents coefficients for the best-fitting model (model 8 response-mapping model).

**Table 5:** Summary of Observed and Predicted Values and Model Performance Statistics for Best-Performing EORTC QLQ-C30 Models

			OLS Model 8		Tobit Model 8		2-Part Model 8		Splining Model 3		ML Model 8	
		Observed Values	Significant Item Levels and Age		Significant Item Levels and Age		Significant Item Levels and Age (P1)		Significant Domain Scores		All Domain Scores and Age/Gender	
Mean (SD)		0.579 (0.342)	0.579 (0.287)		0.579 (0.289)		0.607 (0.300)		0.579 (0.283)		0.573 (0.291)	
Median		0.691	0.650		0.652		0.689		0.646		0.657	
Range		−0.594 to 1	−0.405 to 0.971		−0.394 to 0.946		−0.394 to 0.990		−0.372 to 0.944		−0.338 to 0.942	
MAE			0.139		0.139		0.140		0.143		0.134	
Shrinkage			1.042		1.020		0.940		0.997		1.179	
Model Validation			Mean	MAE	Mean	MAE	Mean	MAE	Mean	MAE	Mean	MAE
Health status (EORTC 29)												
1 (Very poor)	42	−0.006	0.064	0.205	0.064	0.203	0.065	0.195	0.066	0.245	0.047	0.181
2	53	0.176	0.247	0.179	0.243	0.177	0.267	0.185	0.335	0.236	0.226	0.159
3	144	0.429	0.463	0.182	0.460	0.183	0.481	0.184	0.517	0.142	0.452	0.182
4	226	0.622	0.582	0.138	0.582	0.139	0.609	0.141	0.569	0.143	0.583	0.139
5	186	0.718	0.718	0.109	0.721	0.109	0.757	0.107	0.735	0.084	0.709	0.097
6	94	0.832	0.818	0.098	0.820	0.099	0.851	0.104	0.815	0.072	0.814	0.100
7 (Excellent)	26	0.903	0.855	0.080	0.855	0.081	0.893	0.060	0.866	0.134	0.860	0.075
ANOVA		F_6,764_ = 97, *p* < 0.001	F_6,764_ = 114, *p* < 0.001		F_6,764_ = 113, *p* < 0.001		F_6,764_ = 114, *p* < 0.001		F_6,764_ = 117, *p* < 0.001		F_6,764_ = 116, *p* < 0.001	
Mean rank			2.7		2.75		3.2		3.65		2.4	

ECOG, Eastern Cooperative Oncology Group; EORTC QLQ-C30, European Organization for Research and Treatment Quality of Life Questionnaire Core 30; FACT-G, Functional Assessment of Cancer Therapy—General Scale; MAE, mean absolute error; OLS, ordinary least squares.

**Table 6:** Coefficients for Best-Fitting Mapping Model from EORTC QLQ-C30: Response Mapping Model 8

	Mobility	Mobility	Self-Care	Self-Care	Usual Activities	Usual Activities	Pain	Pain	Anxiety/Depression	Anxiety/Depression
Level	2	3	2	3	2	3	2	3	2	3
Physical functioning	−0.0715241	−0.1666518	−0.0492088	−0.0989941	−0.0358454	−0.0851464	−0.0008494	−0.0128045	−0.0143092	−0.0441950
Role functioning	−0.0109798	−0.0066196	−0.0165511	−0.0295817	−0.0321869	−0.0550817	0.0012198	−0.0013792	0.0049731	0.0187775
Emotional functioning	0.0104307	0.0237461	0.0078666	0.0082215	0.0205527	0.0279876	0.0086091	0.0112924	−0.0781099	−0.1475690
Cognitive functioning	−0.0108672	−0.0059660	−0.0098743	−0.0088678	0.0035504	−0.0007108	0.0027021	0.0150750	−0.0065868	0.0056511
Social functioning	0.0030962	0.0109563	−0.0093543	−0.0054659	−0.0213392	−0.0343679	0.0052084	−0.0006402	0.0055038	0.0084157
Fatigue	0.0059733	0.0022788	−0.0220344	−0.0250514	0.0278376	0.0330008	0.0071305	0.0063537	−0.0063396	0.0072863
Nausea and vomiting	0.0005879	0.0157504	0.0068145	0.0186905	0.0218262	0.0215693	0.0054720	−0.0035358	−0.0074123	−0.0088818
Pain	0.0228164	0.0430386	0.0158179	0.0244974	0.0200722	0.0229097	0.1004407	0.1643611	0.0020242	−0.0118933
Dyspnea	0.0016023	0.0044787	−0.0046077	−0.0153410	−0.0053466	−0.0154350	0.0101103	0.0077207	0.0001655	−0.0177905
Sleep disturbance	0.0020489	0.0104134	0.0015579	−0.0001904	−0.0010660	−0.0021797	0.0125753	0.0212104	−0.0029185	0.0116847
Appetite loss	−0.0092890	0.0041667	−0.0001746	0.0095717	−0.0101199	−0.0109212	−0.0127206	−0.0081893	0.0061518	0.0160904
Constipation	−0.0042172	−0.0115196	−0.0041213	−0.0089580	−0.0004575	0.0041718	0.0058912	0.0098999	0.0042562	0.0006725
Diarrhea	−0.0049971	0.0097861	0.0030265	0.0051304	−0.0088893	−0.0111202	−0.0036955	−0.0076847	0.0018030	0.0019909
Financial impact	−0.0012006	−0.0032977	0.0049986	0.0146949	0.0077058	0.0064971	0.0099762	0.0116569	0.0123720	0.0146184
Age	0.0284672	−0.0206177	0.0480864	0.1312050					0.0259679	0.0081053
Female	−0.3486546	−1.3967005								
Constant	3.1686465	3.5415101	0.4980388	−6.6185420	3.4935399	5.6750937	−3.2549790	−9.8187423	4.5615723	6.0238621

EORTC QLQ-C30, European Organization for Research and Treatment Quality of Life Questionnaire Core 30.

## Discussion

This article reports mapping functions from 2 widely used cancer-specific HRQL measures, the EORTC QLQ-C30 and the FACT-G, to the EQ-5D. Generally, OLS and tobit models perform well for both EORTC QLQ-C30 and FACT-G. However, the best-performing model for the EORTC QLQ-C30 was response mapping, whereas OLS gave the best estimates for FACT-G with response-mapping producing poor predictions. The advantage of response mapping being the best model for EORTC QLQ-C30 is that any EQ-5D-3L tariff can be applied to the model. However, these differences in model type are unlikely to represent general findings because it is expected that the best-performing model specification and type can vary by measures mapped to and from, including different EQ-5D-3L country tariffs, the population, and the data set.

The poor performance of the response-mapping approach in the FACT-G data set may be due to the limited number of responders in poor health. This restricted the response-mapping models we fitted to those, including FACT-G overall or domain subscores. The low number of responders in poor health was surprising, because the responders all had stage III or IV cancer and covered a range of different cancers, but it might be due to the FACT-G study asking respondents to fill in a large number of questionnaires. In addition to the ones reported here, responders completed EQ-5D-5L, disease-specific FACT-G modules, and 2 further psychometric questionnaires, making the task quite lengthy and thus potentially biasing the sample to more healthy respondents. The limited range of the FACT-G scores in the estimation data set means that the FACT-G mapping results are not necessarily generalizable to other studies, unless applied to a population in mild to moderate health.

The FACT-G sample size may also have added to the poor performance of response mapping. With a larger sample (e.g., 2000 respondents), you would obtain more accurate predictions of those in level 3, because although the percentage of observations for this level might remain the same (e.g., 3%) the number of observations from which estimates could be made would increase, giving more reliable estimates. Further work is needed on sample size recommendations for the more complex models, such as response mapping.

Other common mapping models are CLAD and GLM. Like the tobit model, the CLAD model also deals with the censored nature of the data and produces consistent estimates in the presence of heteroscedasticity and nonnormality,^35,36^ but it is a median-based model rather than a mean-based model, and there is some debate regarding its suitability for estimating utility values in economic evaluation.^37,38^ Therefore, this model was not fitted here. Generalized linear models (GLMs) were not fitted either because initial GLM models gave similar results to OLS.

A number of mapping functions have been published in the literature for EORTC QLQ-C30.^5,6,8–11,14^ These published studies did not explore the full range of possible mapping functions, as has been done here. Proskorovsky et al.^7^ estimated a mapping function from the same multiple myeloma data set used in this study but, similar to other studies, only reported using OLS. The results here are also based on pooled data from 3 data sets for 3 different types of cancer to produce reliable mapping estimates from a large sample. Pooling 3 types of cancer has increased the generalizability of the results rather than focusing on 1 specific type of cancer. Furthermore, a recent study by Arnold et al.^39^ examined the external validity of published mapping functions from QLQ-C30 to EQ-5D and included the response-mapping function presented in this article. They found that our mapping function performed better than other published mapping functions in predicting EQ-5D.^2,5,6,7,10,12,15,16^ For FACT-G, only 1 published mapping function exists in which the authors acknowledged that estimates were unreliable.^4^

It is evident from the literature that studies report different model fit and selection criteria, with some focusing on model goodness of fit and others on the predictive ability of the model. Mapping models should be selected based on their predictive ability;^25,40^ however, within this, there are still a number of criteria from which a model can be selected, and different choices can result in alternative models being selected. For example, if the accuracy in predicting EQ-5D values based on mean values by severity groups was chosen as the key criterion, then the tobit model would be preferred to the OLS model in the FACT-G models. If focus was on the overall mean for the QLQ-C30 models, then the OLS models would be preferred. In this article, we have given equal weighting to all model-fitting criteria and have used this to generate a ranking of each model. The ranking criteria used here do not take account of the magnitude of the performance statistic and how accurate these are, and further research is needed to explore whether it is possible to account for this when selecting models and to produce more detailed guidelines on selecting appropriate mapping models.

The OLS models perform reasonably well in predicting EQ-5D values from both cancer-specific measures, and response mapping performs the best for the QLQ-C30 data set. We recommend that both types of models are considered for future mapping studies, but note that the response mapping is likely to require a broad spectrum of EQ-5D responses to produce a reliable mapping function and potentially a large number of responses. Both preferred models presented here could be used to predict EQ-5D values in studies that include similar patients; however, the generalizability of the FACT-G mapping function is limited to predictions for respondents in mild and moderate health states. We also recommend transparency in reporting the criteria that are used to select mapping functions that are recommended for use and whether equal weighting is used.

## References

[1] National Institute of Health and Care Excellence (NICE). NICE Guide to the Methods of Technology Appraisal. London: NICE; 2013. 27905712

[2] Longworth L Yang Y Young T Hernandez Alva M Mukuria C Rowen D Use of generic and condition-specific measures of health-related quality of life in NICE decision-making: systematic review, statistical modelling and survey. Health Tech Assessment. 2014;18:9. 10.3310/hta18090 PMC4780954 24524660

[3] Brazier JE Yang Y Tsuchiya A Rowen DL A review of studies mapping (or cross walking) non-preference based measures of health to generic preference-based measures. Eur J Health Econ. 2010 4;11(2):215–25. 19585162 10.1007/s10198-009-0168-z

[4] Cheung YB Thumboo J Gao F Ng GY Pang G Koo WH Mapping the English and Chinese versions of the Functional Assessment of Cancer Therapy General to the EQ-5D Utility Index. Value in Health. 2009 3;12(2):371–6. 18783392 10.1111/j.1524-4733.2008.00448.x

[5] McKenzie L van der Pol M Mapping the EORTC QLQ C-30 onto the EQ-5D instrument: the potential to estimate QALYs without generic preference data. Value in Health. 2009 1;12(1):167–71. 18637140 10.1111/j.1524-4733.2008.00405.x

[6] Khan I Morris S A non-linear beta-binomial regression model for mapping EORTC QLQ-C30 to the EQ-5D-3L in lung cancer patients; a comparison with existing approaches. Health Qual Life Outcomes. 2014;12:163. 25388439 10.1186/s12955-014-0163-7 PMC4234877

[7] Proskorovsky I Lewis P Williams CD Jordan K Kyriakou C Ishak J Mapping EORTC QLQ-C30 and QLQ-MY20 to EQ-5D in patients with multiple myeloma. Health Qual Life Outcomes. 2014;12(1):35. 24618388 10.1186/1477-7525-12-35 PMC4007827

[8] Kontodimopoulos N Aletras VH Paliouras D Niakas D Mapping the cancer-specific EORTC QLQ-C30 to the preference-based EQ-5D, SF-6D, and 15D instruments. Value in Health. 2009 11;12(8):1151–7. 19558372 10.1111/j.1524-4733.2009.00569.x

[9] Wu EQ Mulani P Farrell MH Sleep D Mapping FACT-P and EORTC QLQ-C30 to patient health status measured by EQ-5D in metastatic hormone-refractory prostate cancer patients. Value in Health. 2007 9;10(5):408–14. 17888106 10.1111/j.1524-4733.2007.00195.x

[10] Pickard AS Shaw JW Lin H–W Trask PC Aaronson N Lee TA A patient–based utility measure of health for clinical trials of cancer therapy based on the European Organization for the Research and Treatment of Cancer Quality of Life Questionnaire. Value in Health. 2009;12(6):977–88. 19402850 10.1111/j.1524-4733.2009.00545.x

[11] Crott R Briggs A Mapping the QLQ-C30 Quality of Life Cancer Questionnaire to EQ-5D patient preferences. Eur J Health Econ. 2010 8;11(4):427–34. 20473703 10.1007/s10198-010-0233-7

[12] Crott R Versteegh M Uyl-de-Groot C An assessment of the external validity of mapping QLQ-C30 to EQ-5D preferences. Qual Life Res. 2012 6 29. 10.1007/s11136-012-0220-9 22743734

[13] Versteegh M Leunis A Luime JJ Boggild M Uyl-de Groot CA Stolk EA Mapping QLQ-C30, HAQ, and MSIS-29 on EQ-5D. Med Decis Making. 2012 7;32(4):554–68. 22114301 10.1177/0272989X11427761

[14] Versteegh M Rowen D Brazier J Stolk E Mapping onto EQ-5D for patients in poor health. Health Qual Life Outcomes. 2010;8(1):141. 21110838 10.1186/1477-7525-8-141 PMC3002322

[15] Rowen D Young T Brazier J Gaugris S Comparison of generic, condition-specific, and mapped health state utility values for multiple myeloma cancer. Value in Health. 2012;15(8):1059–68. 23244808 10.1016/j.jval.2012.08.2201

[16] Kim EJ Ko SK Kang HY Mapping the cancer-specific EORTC QLQ-C30 and EORTC QLQ-BR23 to the generic EQ-5D in metastatic breast cancer patients. Qual Life Res. 2012 Sep;21(7):1193–203. 22012023 10.1007/s11136-011-0037-y

[17] Jang RW Isogai PK Mittmann N Bradbury PA Shepherd FA Feld R Derivation of utility values from European Organization for Research and Treatment of Cancer Quality of Life-Core 30 questionnaire values in lung cancer. J Thorac Oncol. 2010;5(12):1953–7. 21155140 10.1097/jto.0b013e3181f77a6a

[18] Kim SH Jo MW Kim HJ Ahn JH Mapping EORTC QLQ-C30 onto EQ-5D for the assessment of cancer patients. Health Qual Life Outcomes. 2012;10:151. 23244763 10.1186/1477-7525-10-151 PMC3542092

[19] Brooks R EuroQol: the current state of play. Health Policy. 1996 7;37(1):53–72. 10158943 10.1016/0168-8510(96)00822-6

[20] Dolan P Modeling valuations for EuroQol health states. Med Care. 1997 11 1;35(11):1095–108. 9366889 10.1097/00005650-199711000-00002

[21] Cella DF Tulsky DS Gray G Sarafian B Linn E Bonomi A The Functional Assessment of Cancer Therapy scale: development and validation of the general measure. J Clin Oncol. 1993 3 1;11(3):570–9. 8445433 10.1200/JCO.1993.11.3.570

[22] Aaronson NK Ahmedzai S Bergman B Bullinger M Cull A Duez NJ The European Organization for Research and Treatment of Cancer QLQ-C30: a quality-of-life instrument for use in international clinical trials in oncology. J Natl Cancer Inst. 1993 3 3;85(5):365–76. 8433390 10.1093/jnci/85.5.365

[23] Pickard AS De Leon MC Kohlmann T Cella D Rosenbloom S Psychometric comparison of the standard EQ-5D to a 5 level version in cancer patients. Med Care. 2007;45(3). 10.1097/01.mlr.0000254515.63841.81 17304084

[24] Greipp PR Miguel JS Durie BGM Crowley JJ Barlogie B Bladé J International staging system for multiple myeloma. J Clin Oncol. 2005 5 20;23(15):3412–20. 15809451 10.1200/JCO.2005.04.242

[25] Brazier J Yang Y Tsuchiya A Rowen D A review of studies mapping (or cross walking) non-preference based measures of health to generic preference-based measures. Eur J Health Econ. 2010;11(2):215–25. 19585162 10.1007/s10198-009-0168-z

[26] Dakin H Review of studies mapping from quality of life or clinical measures to EQ-5D: an online database. Health Qual Life Outcomes. 2013;11(1):151. 24010873 10.1186/1477-7525-11-151 PMC3844400

[27] Huang IC Frangakis C Atkinson MJ Willke RJ Leite WL Vogel WB Addressing ceiling effects in health status measures: a comparison of techniques applied to measures for people with HIV disease. Health Serv Res. 2008 2;43(1 Pt 1):327–39. 18211533 10.1111/j.1475-6773.2007.00745.x PMC2323149

[28] Royston P Sauerbrei W Multivariable modeling with cubic regression splines: a principled approach. Stata J. 2007;7(1):45–70.

[29] Gray AM Rivero-Arias O Clarke PM Estimating the association between SF-12 responses and EQ-5D utility values by response mapping. Med Decis Making. 2006 1;26(1):18–29. 16495197 10.1177/0272989X05284108

[30] Rivero-Arias O Ouellet M Gray A Wolstenholme J Rothwell PM Luengo-Fernandez R Mapping the modified Rankin scale (mRS) measurement into the generic EuroQol (EQ-5D) health outcome. Med Decis Making. 2010;30(3):341–54. 19858500 10.1177/0272989X09349961

[31] Oken MM Creech RH Tormey DC Horton J Davis TE McFadden ET Toxicity and response criteria of the Eastern Cooperative Oncology Group. Amer J Clin Oncol. 1982;5(6). 7165009

[32] Steyerberg EW Eijkemans MJC Harrell FE Habbema JD Prognostic modelling with logistic regression analysis: a comparison of selection and estimation methods in small data sets. Statist Med. 2000;19(8):1059–79. 10.1002/(sici)1097-0258(20000430)19:8<1059::aid-sim412>3.0.co;2-0 10790680

[33] StataCorp. Stata Statistical Software: Release 12. College Station, TX: StataCorp LP; 2011.

[34] Rowen D Brazier J Roberts J Mapping SF-36 onto the EQ-5D index: how reliable is the relationship? Health Qual Life Outcomes. 2009;7:27. 19335878 10.1186/1477-7525-7-27 PMC2683169

[35] Sullivan PW Ghushchyan V Mapping the EQ-5D index from the SF-12: US general population preferences in a nationally representative sample. Med Decis Making. 2006;26(4):401–9. 16855128 10.1177/0272989X06290496 PMC2713176

[36] Powell JL Least absolute deviations estimation for the censored regression model. J Econometrics. 1984;25(3):303–25.

[37] Sullivan PW Are utilities bounded at 1.0? implications for statistical analysis and scale development. Med Decis Making. 2011;31(6):787–9. 22067428 10.1177/0272989X11400755

[38] Pullenayegum EM Tarride JE Xie F O’Reilly D Calculating utility decrements associated with an adverse event marginal Tobit and CLAD coefficients should be used with caution. Med Decis Making. 2011;31(6):790–9. 22067429 10.1177/0272989x11393284

[39] Arnold D Rowen D Versteegh M Morley A Hooper C Maskell N Testing mapping algorithms of the Cancer-Specific EORTC QLQ-C30 onto EQ-5D in malignant mesothelioma. Health Qual Life Outcomes (In Press). 10.1186/s12955-014-0196-y PMC4316600 25613110

[40] Longworth L Rowen D Mapping to obtain EQ-5D utility values for use in NICE health technology assessments. Value in Health. 2013;16:202–10. 23337232 10.1016/j.jval.2012.10.010

